# Healthcare Staff Perceptions Following Inoculation with the BNT162b2 mRNA COVID-19 Vaccine at University Hospitals Coventry & Warwickshire NHS Trust

**DOI:** 10.3390/ijerph18179378

**Published:** 2021-09-06

**Authors:** Tim Robbins, Ioannis Kyrou, Cain Clark, Kavi Sharma, Steven Laird, Lisa Berry, Nina Morgan, Kiran Patel, Sailesh Sankar, Harpal Randeva

**Affiliations:** 1 COVID Clinical Research Department, University Hospitals Coventry & Warwickshire NHS Trust, Clifford Bridge Road, Coventry CV2 2DX, UK; timothy.robbins@uhcw.nhs.uk (T.R.); ioannis.Kyrou@uhcw.nhs.uk (I.K.); cain.clark@uhcw.nhs.uk (C.C.); kavi.sharma@uhcw.nhs.uk (K.S.); Steven.Laird@uhcw.nhs.uk (S.L.); lisa.berry@uhcw.nhs.uk (L.B.); nina.morgan@uhcw.nhs.uk (N.M.); kiran.Patel@uhcw.nhs.uk (K.P.); sailesh.Sankaranarayanan@uhcw.nhs.uk (S.S.); 2 Institute of Digital Healthcare, WMG, University of Warwick, Coventry CV4 7AL, UK; 3 Biomedical Sciences, Warwick Medical School, University of Warwick, Coventry CV4 7AL, UK; 4 Aston Medical Research Institute, Aston Medical School, College of Health and Life Sciences, Aston University, Birmingham B4 7ET, UK; 5 Faculty of Health & Life Sciences, Coventry University, Coventry CV1 5FB, UK

**Keywords:** COVID-19, vaccination, healthcare staff, ethnicity

## Abstract

*Background***:** COVID-19 vaccination programmes offer hope for a potential end to the acute phase of the COVID-19 pandemic. We present perceptions following from a cohort of healthcare staff at the UK NHS hospital, which first initiated the BNT162b2 mRNA COVID-19 (“Pfizer”) vaccination program. *Methods:* A paper-based survey regarding perceptions on the BNT162b2 mRNA COVID-19 vaccine was distributed to all healthcare workers at the University Hospitals Coventry & Warwickshire NHS Trust following receipt of the first vaccine dose. Results: 535 healthcare workers completed the survey, with a 40.9% response rate. Staff felt privileged to receive a COVID-19 vaccine. Staff reported that they had minimised contact with patients with confirmed or suspected COVID-19. Reported changes to activity following vaccination both at work and outside work were guarded. Statistically significant differences were noted between information sources used by staff groups and between groups of different ethnic backgrounds to inform decisions to receive vaccination. *Conclusions:* NHS staff felt privileged to receive the COVID-19 vaccine, and felt that their actions would promote uptake in the wider population. Concerns regarding risks and side effects existed, but were minimal. This research can be used to help inform strategies driving wider vaccine uptake amongst healthcare staff and the public.

## 1. Introduction

The COVID-19 pandemic has resulted in unprecedented challenges to healthcare systems worldwide [1]. Healthcare staff internationally have continued to work under increased pressure throughout the pandemic, coming into contact with large volumes of patients with confirmed or possible COVID-19 [2]. SARS-CoV-2 infection rates among healthcare staff have been shown to be higher compared to that in the general population, with relatively high rates of both serious infections and mortality [2,3,4].

The rapid development [5] and availability of safe and efficacious SARS-CoV-2 vaccines offers the potential promise of providing protection to individuals from both SARS-CoV-2 infection and COVID-19 complications [6,7]. University Hospitals Coventry & Warwickshire (UHCW) NHS Trust was the first site globally to administer the first approved COVID-19 vaccine (BNT162b2 mRNA COVID-19 vaccine, Pfizer/BioNTech: Pfizer Inc.—New Your, NR, USA, and BioNTech—Mainz, Germany) outside a clinical trial on the 8th of December 2020 [8]. In the United Kingdom, priority vaccination status was given to health and social care staff, alongside those aged over 80 and care home residents [9].

There is a long history of healthcare staff vaccination against occupational exposure to a broad range of infectious agents [10,11]. Given the risks and impacts noted for healthcare staff during the COVID-19 pandemic [12,13], vaccination offers an important source of potential protection against SARS-CoV-2 for this at-risk population. Despite the importance of vaccination in the healthcare setting, there has previously been resistance amongst some staff groups/members [14]. As such, there has been recent particular focus on ensuring broad uptake of influenza vaccination amongst healthcare workers alongside understanding healthcare staff perceptions towards vaccination and mechanisms to increase uptake [15,16].

The COVID-19 vaccination effort represents a new and distinct occupational vaccination drive amongst healthcare staff. Prior to the availability of vaccination there were already concerns regarding possible COVID-19 vaccine hesitancy amongst healthcare workers [17,18]. The uptake and response by healthcare workers may also have an important impact on wider society where significant COVID-19 vaccination concerns and misconceptions exist [18]. This research therefore considers healthcare staff perceptions regarding COVID-19 vaccination following receipt of the first dose of the first approved COVID-19 vaccine at UHCW NHS Trust. Indeed, this is the first research study reporting perceptions of those receiving the COVID-19 vaccination and, thus, offers novel insight to the continued COVID-19 vaccine delivery.

## 2. Methods

We conducted a paper-based survey of all staff members at UHCW following receipt of the first dose of the BNT162b2 mRNA COVID-19 vaccine (Pfizer/BioNTech). UHCW is a major tertiary referral centre in the West Midlands region, employing just over 9000 staff, which first initiated the vaccination program with the BNT162b2 mRNA COVID-19 vaccine on the 8th of December 2020. The present survey was conducted between the 11th and 20th December 2020. COVID-19 vaccination was not mandatory for staff at the organization.

The study survey was designed by a multi-disciplinary collaboration of healthcare professionals and was modelled on a previously developed and published survey study considering uptake of COVID-19 antibody testing [19], which provided some validation of the survey questions; however, a fully validated survey for this questionnaire was not available. Ethical approval was granted through the Trust’s COVID-19 ethics committee (GAFREC ID: GF0427). All staff were invited to complete the survey following receipt of the COVID-19 vaccine in a socially distanced format, whilst being monitored for side effects of the administered COVID-19 vaccine. Due to the nature of the present study which invited all eligible staff within the organization, a power calculation to specify a sample size was not conducted prior to the study.

The results were analysed using descriptive and semi-quantitative methods. Descriptive statistics were first computed pertaining to staff role, ethnicity, and survey responses. Due to the nature of the data, non-parametric inferential testing was conducted accordingly. First, data were subjected to the Kruskal–Wallis test, to discern whether differences were evident between staff role/ethnicity and questionnaire responses. In instances where significant differences were identified, subsequent Dwass–Steel–Critchlow–Fligner (DSCF) pairwise comparisons were made, which systematically controls for the family-wise error rate [20]. Analysis was not standardized based on the educational qualification, sex, race or ethnicity of the sample, as this would be difficult to achieve given the sizes of certain groups. All analyses were conducted in R [21], using the Car: (car: Companion to Applied Regression—R package,retrieved from https://cran.r-project.org/package=car (accessed on 19 May 2021)) and nnet: (nnet: Feed-Forward Neural Networks and Multinomial Log-Linear Models—R package, retrieved from https://cran.r-project.org/package=nnet (accessed on 19 May 2021)) packages—[22,23] (Retrieved from https://www.jamovi.org (accessed on 19 May 2021)). Statistical significance was set, a priori, at *p* < 0.05.

## 3. Results

### 3.1. Respondents

Responses were received from 535 UHCW staff members, representing a response rate of 40.9% among the total of 1309 staff members who were vaccinated during the survey period. Among these respondents, 323 (60.3%) reported being from a white British ethnic background, while 208 (38.9%) reported a black or other minority ethnic background (black, Asian and minority ethnic, BAME, background). The job roles of study respondents are presented in Table 1. Moreover, 25 staff members (4.6%) reported being part of an extremely clinically vulnerable (“shielding”) group, whilst 72 (13.5%) reported that they had received a previously positive antibody test, and 98 (18.3%) reported that they had not previously had an antibody test. Finally, 385 respondents (71.9%) reported having a primary clinical degree, with 279 of those (72.5%) reporting that this degree had been awarded in the United Kingdom.

### 3.2. Perceptions of Receiving the First Dose of the BNT162b2 Mrna COVID-19 Vaccine

In total, 519 respondents (97.0%) reported that they felt privileged to have early access to this COVID-19 vaccine. Moreover, 514 (96.2%) reported that having a COVID-19 vaccine as a healthcare staff member would “reassure the general public about the safety and effectiveness of receiving a COVID-19 vaccine”.

**Figure 1:**
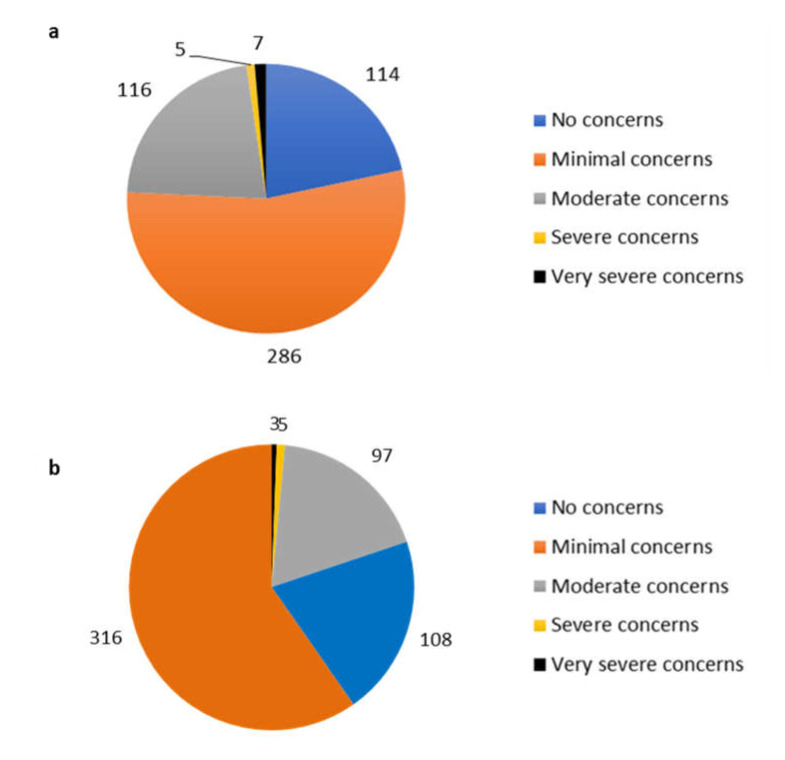
Concern regarding BNT16b2 mRNA COVID-19 vaccine: **1a**= safety and long term risks, **1b** = side effects.

Notably, 414 respondents (77.4%) reported having some concerns regarding the safety and long-term risk of this vaccine; however, the vast majority were reported as “minimal” concerns. In addition, 421 (78.7%) reported some concerns regarding side effects from this vaccine, with the vast majority of concerns being reported in the “minimal” corresponding category. Figure 1 presents a breakdown of these reported concerns.

### 3.3. Behaviour Following COVID-19 Vaccination

In total, 248 respondents (46.4%) stated that they minimised the amount of time they spent in close contact with patients diagnosed or suspected of having COVID-19, and, of these staff, 113 (45.6%) reported that they would be happy to have greater interaction with such patients following the COVID-19 vaccine. Furthermore, 186 (34.8%) reported that as a consequence of the COVID-19 pandemic they minimised time spent in close contact with all patients, and, of these staff, 101 (54%) reported that they would be happy to have greater interaction with patients following the COVID-19 vaccine.

**Figure 2:**
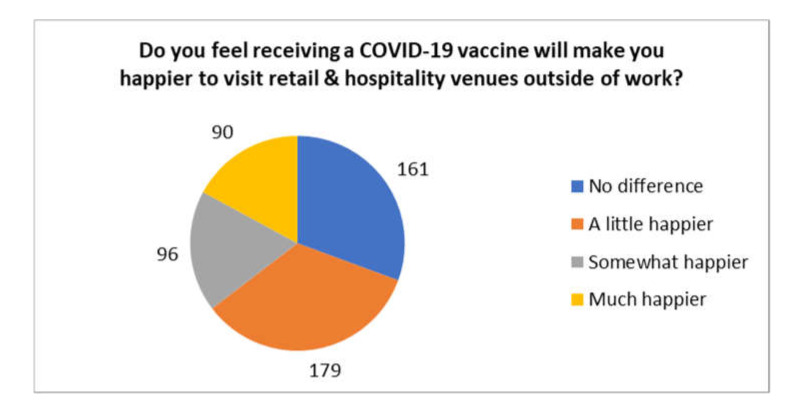
NHS staff perceptions regarding visiting hospitality and retail.

Overall, 414 of the respondents (77.4%) reported that they felt that this vaccination would make no difference to the wearing of personal protective equipment (PPE) at work, with 116 (21.7%) reporting that PPE would be less important, and 18 (3.4%) feeling that PPE would be much less important at work. Furthermore, 397 (74.2%) reported that they felt that this vaccination would make no difference to wearing PPE outside of work, with 125 (23.4) feeling it would be less important. Finally, 365 (68.2%) reported that they would feel happier to see their families having received this vaccination for COVID-19, while 165 (30.1%) reported that receiving a COVID-19 vaccine would make no difference to how happy they were to visit hospitality and retail venues outside of work. The full breakdown of responses to this question is presented in Figure 2.

### 3.4. Staff Burnout

In total, 162 (30.3%) of the NHS staff members in this survey reported they felt “anxious and burnout out at work” often or all of the time. Moreover, 289 (54.0%) reported that receiving the COVID-19 vaccination would reduce their feelings of anxiety or burnout whilst at work. Only 36.7% of the staff who reported anxiety and burnout “all of the time” felt that the vaccine would reduce their feelings of anxiety or burnout, in comparison to 47.9% of the staff members who reported feelings of anxiety and burnout only rarely.

### 3.5. Sources of Information to Support Decision Making Regarding the COVID-19 Vaccination

Table 2 presents the responses to the survey question regarding how important different information sources were in making the decision to have a COVID-19 vaccine. Overall, academic publications were seen as the most important source of information; however, significant and important differences were noted between staff groups/roles and between ethnic backgrounds. Medical doctors reported academic publications to overwhelmingly be the most important information source, whereas for allied health professionals this was information from other NHS staff. Furthermore, NHS Trust-communicated information was felt to be most important for nurses. Statistically significant differences (*p* < 0.05) were noted particularly for use of social media (Facebook) where DSCF post-hoc testing showed significant differences between doctors and health care assistants (HCAs) and academic publications where DSCF post-hoc testing showed significant differences between: allied healthcare and HCAs; medical doctors and HCAs; nurses and HCAs.

Furthermore, statistically significant differences regarding the importance of different information sources for making the decision to have a COVID-19 vaccine were also noted with regards to ethnic background. In particular, significant differences were noted between respondents of BAME and White background for Twitter (*p* < 0.05), Facebook (*p* < 0.05), and NHS colleagues (*p* < 0.05) (Table 2).

## 4. Discussion

The COVID-19 pandemic has resulted in unprecedented challenges both to healthcare services and to the wider society in the UK and worldwide [24]. Particularly, healthcare staff have been under immense pressure to manage high volumes of patients with COVID-19, and also at risk of exposure to SARS-CoV-2 infection [25]. Approved COVID-19 vaccines now offer the promise of potentially reducing the rates of SARS-CoV-2 infection and severe COVID-19 [6,7], and are particularly important to healthcare staff [26]. Notably, certain healthcare staff groups, in particular those of BAME background, appear to have been disproportionately affected by severe COVID-19 [4]. Moreover, historically, there has been reluctance amongst healthcare staff to receive occupational related vaccines, particularly the influenza vaccine [27]. Therefore, the present research offers novel insight to the perceptions of healthcare staff receiving the COVID-19 vaccination.

During the COVID-19 pandemic the workload on healthcare staff has been substantial, and it is reassuring to document that NHS staff members felt privileged to have access to the first approved COVID-19 vaccine early in the vaccination process. This is particularly important on the background of the large proportion of NHS staff (almost one third) reporting feelings of anxiety and burnout. The latter finding is similar to that of other studies reporting the impact of this pandemic on staff burnout [28,29]. Some of these feelings of anxiety and burnout may be attributed to fear of contracting and spreading SARS-CoV-2, including to family members, and of the subsequent impact of COVID-19, including on family finances [30]. Of note, this is the first study identifying that for many NHS staff members receiving a COVID-19 vaccine, this vaccination reportedly reduced perceptions of anxiety and burnout. This is an important factor in supporting wider wellbeing provisions for healthcare staff. However, this reported reduction regarding burnout did not apply to all respondent NHS staff members, and those reporting the greatest levels of burnout were less likely to associate receiving a COVID-19 vaccine with reduced feelings of anxiety/burnout. The levels of worry regarding side effects (78.7%), including a small proportion expressing high levels of concern, was potentially surprising, despite these being people who opted to receive vaccination. This could possibly indicate that fear of COVID-19 outweighs the fear of side effects from COVID-19 vaccination; however, this raises important questions for those who haven’t received vaccination and whether they have high levels of concern that may indeed negatively be influencing their decision making regarding receiving a COVID-19 vaccine. This is an important area for future research.

Responding to the risks associated with occupational exposure to COVID-19, a proportion of the NHS staff in this study reported minimising their contact both with patients with confirmed or suspected COVID-19 and those not suspected to have COVID-19. COVID-19 vaccination would result in approximately half of these staff being prepared to have greater interactions with either patient category. This is concerning and merits further research, as the healthcare provider-patient relationship may be adversely affected by NHS staff keeping a greater distance from patients and not being reassured by the COVID-19 vaccine rollout.

Moreover, the present survey identified additional important aspects regarding behaviour change. Particularly, NHS staff broadly did not feel that this vaccination would facilitate a reduction in the importance of PPE, albeit there was a small subset who felt a reduction in the use of PPE was warranted. The latter needs to be urgently addressed by NHS Trusts, potentially with targeted education and reinforcing the importance of PPE despite having received a COVID-19 vaccine. Indeed, perceptions around the post-vaccine role of PPE are vitally important, given that the risks of SARS-CoV-2 transmission despite vaccination remain uncertain. Furthermore, in relation to wider society, it was perhaps concerning that approximately a third of the respondents did not feel that receiving a COVID-19 vaccine would give them increased confidence to visit leisure and retail facilities. This finding among healthcare workers may represent an early sign that it may take longer for the COVID-19 vaccination program to contribute to the recovery of the broader economy and to inspire confidence for the wider public to visit leisure and retail facilities.

Another key important finding of the present research relates to the differences between information sources used to inform decisions for having this vaccination. Interestingly, the present findings note substantial differences regarding this point between NHS staff members of different staff roles and ethnic backgrounds. To that respect, it is important that information provided to support individuals to receive a COVID-19 vaccine are further tailored/targeted to the information sources that they identify as being most important for this decision. As it is possible that there will be a need for repeating the COVID-19 vaccination, targeting the relevant information appropriately becomes a key point for the success of such programs in the following months/years.

Since the delivery of our survey, a number of other research studies have reported outcomes internationally. In France, a survey was completed ahead of a vaccine becoming available, which showed that the majority would receive vaccination should it be an option; however, this survey also noted similar differences between staff groups [31]. A further multi-centre survey in France and French speaking parts of Belgium and Canada demonstrated particularly that hesitancy was driven by concerns regarding vaccine safety—a sentiment observed in our study [32,33]. Biswas et al. explored this further, noting that a combination of safety concerns, efficacy concerns and side effect concerns was a major driver of vaccine hesitancy, and stressing the importance of education [33]. Qattan et al. further observed that perceived high risks of infection and a belief in compulsory vaccination were positive drivers of vaccination, representing interesting areas of further research in relation to the UK survey presented here [34]. Overall, we show relatively similar sentiments amongst UK healthcare workers in comparison to those reported internationally, with additional detail provided in our survey looking at the impact of ethnicity and behaviour change.

There are a number of strengths to the research presented here, which we believe covers an important and currently under-researched area of the COVID-19 vaccination program. Indeed, this is the first—to our knowledge—survey focused on the detailed perceptions of NHS staff following inoculation with the first approved COVID-19 vaccine at UHCW. Despite the ongoing pressures on NHS healthcare staff/services, a good response rate was achieved for this study, whilst a broader range of relevant questions were included, providing data which underwent a rigorous statistical analysis. However, there are also certain study limitations. Particularly, since this represents a single centre study, it is possible that the present results cannot necessarily be extrapolated to all NHS hospitals nationally. Moreover, the sample size of this single centre study did not allow further subgroup analyses. Indeed, it would be important to repeat the survey at more than one hospital site, and this could be particularly relevant in the context of booster vaccines currently being proposed in the UK. A follow-up of this survey could also further consider differences between population groups based on both age and gender, which wasn’t included within the present survey. Moreover, the nature of this survey-based perception research is not able to capture inadvertent or unconscious factors driving the relevant decisions of staff members that participated in the survey, whilst these respondents may also not actually go on to act in the manner they report that they anticipate. Moreover, the present results should be interpreted in the context that the respondents were staff members who opted to have the COVID-19 vaccination, and should not therefore be extrapolated to those choosing not to receive a COVID-19 vaccine. However, it is plausible that, over time, the actions and perceptions of those that had a COVID-19 vaccine may influence those with greater hesitancy.

In conclusion, we believe that this work suggests that actions are needed with regards to targeting more tailored information regarding the COVID-19 vaccination to different NHS staff groups and demographics. To this aim, there is a strong argument for evaluation of social media content in relation to COVID-19 vaccine relevant information in order to better understand how this information is presented in such social media environments. In addition, we suggest that further research work is needed to explore how NHS staff actions change in relation to having received a COVID-19 vaccine (e.g., in relation to PPE). We hope that this initial work, alongside the further work proposed here, can be used to ensure that NHS staff are given the appropriate information that is required to make confident, well-informed decisions regarding the COVID-19 vaccination, and to take sensible actions and precautions after receiving this vaccination.

## 5. Conclusions

In conclusion, this work identifies that NHS staff felt privileged to receive the COVID-19 vaccine, and felt that their actions would promote uptake in the wider population. Staff did however raise concerns regarding risks and side effects, but these were minimal. We feel this research can be used to help inform strategies driving wider vaccine uptake amongst healthcare staff and the public and may be particularly important if future booster doses of vaccine are needed in the future.

## Figures and Tables

**Table 1:** Roles of healthcare staff respondents.

Role	*N*	%
Nurse/midwife	189	35
Medical doctor	169	32
Healthcare assistant	71	13
Allied healthcare professional	59	11
Patient facing admin/suppport	21	4
Non-patient facing support	12	2
Other/not specified	8	1
Manager	6	1

**Table 2:** Importance of information source regarding the decision to receive the COVID-19 vaccination based on staff group/role and ethnic background.

*Information Source Regarding Decision to Receive the COVID-19 Vaccination*						
***Response: Very Important***	***Traditional News Sources***	***Social Media (Twitter)***	***Social Media (Facebook)***	***NHS Colleagues***	***UHCW-Communicated Info***	***Academic Publications***
**% (number) per NHS group/role:**						
**Allied healthcare professionals**	24.5% (13)	7.5% (4)	11.8% (6)	73.2% (41)	48.1% (26)	69.2% (36)
**Medical doctor**	30.2% (48)	8.2% (12)	7.5% (11)	51.9% (84)	44.3% (70)	83.3% (135)
**Healthcare assistant**	33.3% (20)	9.9% (7)	12.0% (6)	60.3% (35)	55.6% (30)	41.5% (22)
**Nurse**	31.7% (53)	10.7% (12)	7.9% (13)	61.4% (105)	64% (110)	77.8% (129)
***Response: Not important***	***Traditional news sources***	***Social media (Twitter)***	***Social media (Facebook)***	***NHS Colleagues***	***UHCW-communicated info***	***Academic publications***
**% (number) per NHS group/role:**						
**Allied healthcare professionals**	37.7% (20)	75.5% (40)	78.4% (40)	3.6% (2)	9.3% (5)	5.8% (3)
**Medical doctor**	27.0% (43)	79.6% (117)	82.3% (121)	12.3% (20)	13.9% (22)	5.6% (9)
**Healthcare assistant**	16% (10)	46.5% (33)	60.0% (30)	6.9% (4)	1.9% (1)	17.0% (9)
**Nurse**	21.6% (30)	53.1% (60)	73.9% (122)	5.3% (9)	2.9% (5)	4.2% (7)
***Response: Very important***	***Traditional news sources***	***Social media (Twitter)***	***Social media (Facebook)***	***NHS Colleagues***	***UHCW-communicated info***	***Academic publications***
**% (number) per NHS group/role:**						
**BAME**	40.9% (70)	18.5% (29)	13.0% (20)	63.1% (113)	60.2% (103)	77.2% (131)
**White**	27.6% (88)	4.1% (12)	4.4% (13)	58.0% (182)	51.8% (162)	70.9% (214)
***Response: Not important***	***Traditional news sources***	***Social media (Twitter)***	***Social media (Facebook)***	***NHS Colleagues***	***UHCW-communicated info***	***Academic publications***
**% (number) per NHS group/role:**						
**BAME**	21.6% (37)	54.8% (86)	60.1% (92)	6.1% (11)	7.0% (12)	4.1% (7)
**White**	24.8% (79)	84.5% (246)	84.3% (247)	7.2% (30)	2.9% (9)	8.6% (26)

## Data Availability

No additional data is available alongside this study, as a consequence of the local ethical approval process above.
